# Improved detection of group B *Streptococcus* in rectovaginal samples of pregnant individuals in Brazil using chromogenic medium

**DOI:** 10.1128/spectrum.02459-25

**Published:** 2025-12-23

**Authors:** Natalia Silva Costa, Andre Rio-Tinto, Julia de Deus Santana, Julia Ferrarezi Favorato Moriel Garcia, Isabella Bittencourt Ferreira Pinto, Laylla Graca Barros, Ana Clarisse Merces, Eduardo Oliveira Bressan, Laura Maria Andrade Oliveira, Sergio Eduardo Longo Fracalanzza, Lucia Martins Teixeira, Penélope Saldanha Marinho, Tatiana Castro Abreu Pinto

**Affiliations:** 1 Instituto de Microbiologia Paulo de Goes, Universidade Federal do Rio de Janeiro 28125, Rio de Janeiro, Brazil; 2 Departamento de Microbiologia, Imunologia e Parasitologia, Faculdade de Ciências Médicas, Universidade do Estado do Rio de Janeiro, Rio de Janeiro, Brazil; 3 Laboratório de Enterobactérias, Instituto Oswaldo Cruz, FIOCRUZ, Rio de Janeiro, Brazil; 4 Maternidade Escola, Universidade Federal do Rio de Janeiro 28125, Rio de Janeiro, Brazil; Emory University School of Medicine, Atlanta, Georgia, USA

**Keywords:** group B *Streptococcus*, pregnant individuals, chromogenic media

## Abstract

Universal screening of pregnant individuals followed by intrapartum antibiotic prophylaxis is the most effective approach to prevent group B *Streptococcus* (GBS) neonatal infections. Traditional protocols for GBS detection in rectovaginal samples involve a pre-enrichment step using selective broth, followed by inoculation onto blood agar, but these methods can be labor-intensive, time-consuming, and costly in low-resource settings. Here, we compared the performance of conventional selective broth prior to plating (THB-NAG) with direct plating onto chromogenic media (CA) for GBS detection among 1,128 pregnant individuals in Brazil. Overall, GBS was detected in 7.8% (88) of samples. CA detected a significantly (*P* < 0.0001) higher number of positive samples (6.5%; 73) when compared to THB-NAG (2.7%; 31). CA was also superior in inhibiting contaminant microbiota, was faster and more cost-effective, and showed high sensitivity and accuracy levels when compared to THB-NAG, although it had failed when samples had low GBS density. Adopting more feasible and cost-effective protocols, such as the CA method shown in this study, can lead to improved adhesion to the universal antenatal GBS screening and thus, to the prevention of early GBS disease in low-resource settings, such as Brazil.

**IMPORTANCE:**

Group B *Streptococcus* (GBS) is a leading cause of life-threatening neonatal infections. The recommended strategy to prevent GBS disease is based on GBS screening of pregnant individuals, followed by antibiotic prophylaxis. However, the standard laboratory method for GBS detection requires extended incubation time and may present challenges associated with costs and feasibility, making it difficult for routine implementation in low-resource settings. Additionally, the overgrowth of accompanying microbiota can hinder GBS detection, potentially reducing the method’s sensitivity. This creates barriers to effective prevention strategies. In this context, chromogenic media have emerged as a promising alternative, offering faster, simpler, and more accurate detection through color-based differentiation of colonies. However, this method has not been fully evaluated in local healthcare conditions in countries like Brazil. This study was undertaken to investigate whether chromogenic media could provide a more practical, affordable, and reliable option for GBS detection in routine prenatal care.

## OBSERVATION

*Streptococcus agalactiae* (group B *Streptococcus*, GBS) is a major cause of neonatal infections, with mortality rates of up to 50% (1). Vertical transmission is the main route for neonatal contamination, since GBS colonizes the rectovaginal area of 10%–40% of pregnant individuals worldwide (2). For preventing early GBS neonatal disease, universal screening of pregnant individuals, followed by intrapartum antibiotic prophylaxis (IAP) for those who test positive for GBS, is the most effective approach (3, 4). Conventional culture-based techniques using selective enrichment broth remain the recommended standard for antenatal GBS screening (5). Although this approach has been demonstrated to enhance GBS detection (6), incubation in selective broth prior to plating is time-consuming, requiring up to 72 h to yield results, and may present challenges associated with costs and feasibility, particularly in low-resource settings. GBS screening is usually performed at 36th–37th gestational weeks (3). However, reducing turnaround time (TAT) is particularly relevant because faster methods allow screening to be performed closer to delivery. Since GBS colonization is intermittent, testing nearer to birth increases the likelihood of accurately identifying colonized women. Consequently, rapid detection may support the appropriate use of IAP, ensuring it is administered to women who are truly colonized at the time of delivery. This is especially important in low-resource settings, where delays in laboratory results and patient follow-up can compromise effective prevention.

Chromogenic media, in turn, can differentiate specific microorganisms even in mixed culture conditions by colony color, in a fast and cost-effective way. Although they have been available for GBS detection in rectovaginal samples for more than 10 years, a comprehensive evaluation of chromogenic media performance has not been performed in our low-resource setting. In Brazil, routine prenatal care still lacks standardized GBS screening protocols, but the most common approach is the use of selective enrichment broth, followed by plating onto blood agar (7). Here, we evaluated the GBS detection in rectovaginal samples of pregnant individuals at 35th–37th gestational weeks by plating onto chromogenic media directly and compared it to the conventional method based on prior enrichment using selective broth. Although international guidelines were updated in 2020 to recommend screening at 36th–37th weeks, we have maintained the range of 35–37 weeks for consistency and comparability with previous years of data.

We analyzed 1,128 rectovaginal samples collected from pregnant individuals at 35–37 gestational weeks attended at Teaching Maternity of UFRJ, in Rio de Janeiro, Brazil, from September 2021 to December 2024. Rectovaginal samples were collected during routine antenatal care using the combined swab method (5), employing flocked swabs (Copan Diagnostics Inc., USA) that were kept in Amies transport medium until processing. The project was approved by the local research ethics committee under number 43389321.9.0000.5257, and written informed consent was obtained from all participants. Each rectovaginal sample was subjected to two procedures in parallel: (i) THB-NAG method (7): involving a pre-enrichment step using Todd-Hewitt broth (THB; Sigma-Aldrich, USA) supplemented with nalidixic acid (15 μg/mL; Sigma-Aldrich) and gentamicin (8 μg/mL; Sigma-Aldrich) incubated under 5% CO_2_ atmosphere for 18–24 h at 37°C, followed by plating onto 5% sheep blood agar plates (PlastLabor, Brazil); and (ii) CA method: consisting of directly streaking onto CHROMagar StrepB (CHROMagar, France) and incubating for 24 h at 37°C under conventional atmosphere. Identification of beta-hemolytic colonies grown on blood agar and mauve-colored colonies on chromogenic medium was performed by MALDI-TOF MS (Bruker Microflex LT, Germany), according to the manufacturer’s instructions. Statistical analysis was performed using the Chi-square test with the support of GraphPad Prism software version 5 (GraphPad Software, USA); *P* values of <0.05 were considered statistically significant. Sensitivity, specificity, accuracy, positive predictive value, and negative predictive value were calculated using the online tool MedCalc version 23.1.7 (8). The total number of positive subjects, combining results of both methods (THB-NAG and CA), was considered 100% and used as the reference standard for these calculations.

Overall, considering both approaches, GBS was detected in 7.8% (88/1128) of rectovaginal samples investigated. GBS occurrence was lower than that reported in previous studies in Brazil (7, 9, 10); however, we have recently shown a trend of reduced maternal GBS carriage rates in our setting in the last years, especially after the COVID-19 pandemic (11). Although this cohort showed a relatively low rate of positivity (around 8%), the study was carried out with a large population size (more than 1,000 participants) and comprised a large timeframe (more than 3 years).

When analyzed alone, the CA method performance was highly superior (*P* < 0.0001), yielding 6.5% (73/1,128) of positivity against 2.7% (31/1,128) of the THB-NAG method. Among 88 GBS-positive samples, only 16 (18.2%) were detected in both approaches; the other 72 were either exclusively detected by the THB-NAG method (15) or, in its majority, by the CA method only (57). Despite the notable specificity of 100% and accuracy of 94.9%, the THB-NAG method showed a significantly lower sensitivity of 35.2%, suggesting that it may miss a critical proportion of positive samples. CA method showed high sensitivity (82.9%), specificity (100%), and accuracy (98.7%) levels (Table 1), similar to what has been previously reported in other places (12).

**TABLE 1:** Performance of direct CA plating against THB-NAG enrichment broth subcultured on blood agar for GBS screening

	Sensitivity	Specificity	Accuracy	PPV^*a*^	NPV^*b*^
THB-NAG	35.2% (95% CI^*c*^, 25.3% to 46.1%)	100% (95% CI, 99.6% to 100%)	94.9% (95% CI, 93.5% to 96.1%)	100% (95% CI, 88.8% to 100%)	94.8% (95% CI, 94% to 95.5%)
CA	82.9% (95% CI, 73.4% to 90.1%)	100% (95% CI, 99.6% to 100%)	98.7% (95% CI, 97.8% to 99.2%)	100% (95% CI, 95.1% to 100%)	98.6% (95% CI, 97.8% to 99.1%)

^*a*^ PPV, positive predictive value.

^*b*^ NPV, negative predictive value.

^*c*^ 95% CI, confidence interval.

Among 57 false-negative samples in the THB-NAG method, most of them (47) yielded beta- or alpha-hemolytic colonies identified as *Enterococcus* sp., *Staphylococcus* sp., or other *Streptococcus* species, while the remaining (10) had non-hemolytic colonies or no growth, and thus, were not identified. Many rectovaginal samples have *Enterococcus* spp. and/or *Staphylococcus* spp. as accompanying microbiota, and overgrowth of such bacteria can impair or disguise GBS growth or visualization of GBS colonies on blood agar plates (13), which may have been the case for 47 false-negative samples yielded by THB-NAG. Furthermore, our protocol followed the routine practice adopted in most laboratories worldwide, where beta-hemolytic colonies are typically selected for further analyses. Although non-hemolytic GBS strains have been isolated from pregnant individuals and from cases of invasive disease in neonates and adults (14–16), they are relatively rare, comprising only about 5%–6% of strains in screening specimens (5), which supports this usual practice internationally. We acknowledge that limiting the analysis to beta-hemolytic colonies may miss these uncommon non-hemolytic strains, which could partly explain the lower sensitivity observed with the conventional method. However, in our study, among the 57 false-negative samples in the THB-NAG method, only three were non-hemolytic, differing from the typical beta-hemolytic pattern. This finding suggests that overlooking non-beta-hemolytic GBS strains had little impact on the overall performance of the method. It is worth noting that this selection criterion reflects procedures routinely applied worldwide, indicating that such methodological limitations are likely shared across most studies employing the conventional culture-based approach. While non-beta-hemolytic strains could be disregarded when using the THB-NAG method, they yield expected colony color in the CA method.

Among 15 false-negative samples in the CA method, 11 did not yield expected mauve-colored colonies, and, as such, were not identified, while the remaining four had mauve colonies identified as other bacterial species, including *Aerococcus* sp., *Lactobacillus* sp., *Streptococcus anginosus,* and *Streptococcus mitis/oralis*. While the CA method showed a superior performance regarding inhibition of typical contaminant microbiota, the higher selective and restrictive feature may also have impaired GBS detection in samples with low GBS density, which could have been the case for the 15 false-negative samples in this approach.

Ideally, combining both approaches (THB-NAG and CA) would increase the likelihood of recovering GBS from clinical samples, allowing GBS detection even in low densities and among heavily contaminated samples (17). However, in low-resource settings with limited financial and structural resources, this is not feasible. It seems, however, that the best approach varies according to the setting. While previous studies have shown that sensitivity can be substantially improved with the use of selective enrichment broth (6), others have demonstrated that the performance of the CA method is not inferior to the selective broth enriched approach (13, 18). Here, we showed that the CA method, when compared to the THB-NAG method, presented higher sensitivity (82.9% versus 35.2%) and accuracy (98.7% versus 94.9%), suggesting that the former would be a better option for our setting.

In addition to a superior performance for detecting GBS, the CA method was also faster. The TAT for the conventional THB-NAG method was around 45 h, while the TAT for the CA method was around 25 h. In terms of costs, although blood agar alone is generally less expensive than chromogenic agar, the overall cost per sample in our context, considering consumables, was comparable between the two methods, since the THB-NAG method required the combined use of enrichment broth supplemented with antibiotics, blood agar, and a CO_2_ incubator, which can be a relatively expensive equipment. To improve the adhesion to GBS screening, especially in low-resource settings, reducing the associated TAT and costs is an urgent and constant need.

In summary, the CA method was able to detect a significantly higher number of positive samples, was able to more efficiently inhibit accompanying microbiota, was faster and more cost-effective, and showed high sensitivity and accuracy levels, but had performance limitations when the samples had low GBS densities. Overall, the CA method, when compared to THB-NAG, is shown to be a more efficient and time-saving alternative for our low-resource setting, and its adoption could help to improve the national adherence to the universal antenatal screening protocol, and thus, contribute to the prevention of early GBS disease in Brazil.
